# Physiological and Genomic Characterization of Oligotrophic *Nitrobacter* Isolated from a Forest Soil in Japan

**DOI:** 10.1264/jsme2.ME24114

**Published:** 2025-06-17

**Authors:** Yoichiro Kobayashi, Takuya Ninomiya, Yuki Shiraishi, Ayano Kaneko, Megumi Kuroiwa, Yuichi Suwa, Hirotsugu Fujitani

**Affiliations:** 1 Department of Biological Sciences, Chuo University, Japan

**Keywords:** genome, isolation, kinetics, nitrification, *Nitrobacter*

## Abstract

Nitrite is a key intermediate in global nitrogen cycles. It has been widely recognized that the accumulation of nitrite is often not appreciable in environments, and nitrite concentrations in canonical media for the cultivation of nitrite-oxidizing bacteria (NOB) in laboratories may not be low enough to recover oligotrophic NOB. We herein report the isolation, physiology, and genomics of oligotrophic NOB from a Japanese forest soil. NOB in soil samples were enumerated using the most probable number method with a medium containing urea for enriching oligotrophic NOB. Urea was completely converted into nitrate, and nitrite was not detected in any nitrifier-positive tubes cultivated after 9‍ ‍weeks of incubation. After subculturing NOB several times in a medium supplemented with 1‍ ‍mM nitrite and performing the extinction-dilution procedure, a novel strain oxidizing nitrite to nitrate was obtained and designated as strain CN101, which was affiliated with the genus *Nitrobacter* at the 16S rRNA gene level. The half-saturation constant of strain CN101 was lower than other known *Nitrobacter* strains, suggesting that *Nitrobacter* strains do not always exhibit low affinity for nitrite. The complete genome of strain CN101 included a larger number of nitrite/nitrate transporters than other *Nitrobacter* strains, which may serve as tools for flexibly adapting to varying nitrite concentrations in soils. Therefore, the physiological and genomic characteristics of strain CN101 will expand knowledge of the ecologically important but understudied genus *Nitrobacter*.

Nitrite-oxidizing bacteria (NOB) catalyze the oxidation of nitrite to nitrate, the second step of nitrification and a key reaction in the biogeochemical nitrogen cycle. The phylogenetic diversity of NOB has become more complex, including the classes *Alphaproteobacteria*, *Betaproteobacteria*, and *Gammaproteobacteria* and phyla *Nitrospirae*, *Nitrospinae*, and *Chloroflexi* (Spieck *et al.*, 2020). A new bacterial phylum with nitrite-oxidizing potential in oligotrophic marine sediments, tentatively named *Candidatus* Nitrosediminicolota, was recently reported through genome reconstruction and phylogenetic ana­lyses (Zhao *et al.*, 2024). A member of the genus *Nitrobacter* affiliated with *Alphaproteobacteria* has been recognized as a model NOB since its first discovery by Sergei Winogradsky. To date, *Nitrobacter* has been cultivated and isolated from a number of different environments (Hankinson and Schmidt, 1988; Bock *et al.*, 1990; Sorokin *et al.*, 1998). The variety of sample sources for *Nitrobacter* isolates demonstrates that the functional guild is distributed widely and may be adapted to a broad range of environmental conditions (Daims *et al.*, 2011). Acidic conditions are generally unfavorable for most NOB, whereas some *Nitrobacter* isolates obtained from soils are moderately acidophilic or acidotolerant NOB (de Boer *et al.*, 1991; Endo *et al.*, 2024; Hink *et al.*, 2024). Additionally, some *Nitrobacter* strains showed varying levels of growth in the presence of organic matter (Steinmüller and Bock, 1976). The recently published whole or draft genome sequences of *Nitrobacter* species revealed genomic diversity and metabolic potentials allowing for adaptation to the varying environmental conditions described above (Starkenburg *et al.*, 2006, 2008; Mellbye *et al.*, 2017; Soghomonian *et al.*, 2024). Since ecological flexibility is high within the genus *Nitrobacter*, it is important to isolate and characterize more *Nitrobacter* strains in order to obtain a more detailed understanding of microbial ecology in the nitrogen cycle.

The concentration of nitrite, as an energy source of NOB, is often not sufficiently high in their habitat; generally ≥2‍ ‍μM in soil and no higher than 1.08‍ ‍mM (van Cleemput and Samater, 1996). This may be explained by the immediate conversion of nitrite to nitrate by NOB. Therefore, NOB, the affinity of which for nitrite is high, could scavenge nitrite as soon as it is supplied to their habitat by ammonia oxidation. Previous studies demonstrated that *Nitrospira* species isolated from activated sludge and freshwater lakes generally exhibited high affinity for nitrite (Nowka *et al.*, 2015a; Ushiki *et al.*, 2017; Fujitani *et al.*, 2020). The affinity for nitrite of isolates categorized to the genus *Nitrobacter* was typically lower (Nowka *et al.*, 2015a). As described above, predominant soil NOB mostly have an oligotrophic nature. Although the concentration of nitrite added to inorganic media used to isolate and cultivate *Nitrospira* strains is low (0.14–2.9‍ ‍mM) (Lebedeva *et al.*, 2008; Lebedeva *et al.*, 2011; Ushiki *et al.*, 2013; Fujitani *et al.*, 2014, 2020; Nowka *et al.*, 2015b), it is still markedly higher than that found in soil environments and may not be low enough to recover oligotrophic NOB. Therefore, a continuous supply of nitrite to NOB at a low concentration may be key for obtaining ecologically meaningful NOB isolates from environments.

In the present study, instead of adding nitrite to the medium at a higher concentration, 0.75‍ ‍mM urea was supplemented to mineral minimum medium for a nitrifying community including ammonia-oxidizing microorganisms (AOMs) and NOB. Based on our previous study (Endo *et al.*, 2024), urea is gradually hydrolyzed to ammonia by a urea decomposer, and this is followed by continuous nitrite production by AOMs. Since NOB were fed with nitrite at low concentrations in this community, it was expected to cultivate oligotrophic NOB. Additionally, we selected a forest soil as a sample source because pure cultures of NOB from soil environments are extremely limited. Therefore, the present study 1) investigated whether oligotrophic NOB may be isolated from soil using the strategy stated above, which includes an assessment of their affinity for nitrite, and 2) compared the physiology and genomics of previously identified *Nitrobacter* isolates.

## Materials and Methods

### Sample

Soil samples were collected at the Fukuroyamazawa test site (Fig. S1) of the University of Tokyo in Kamogawa city, Chiba, on July 29, 2016. The sampling site was previously investigated to characterize organic and surface soil layers (0–10‍ ‍cm) in forest soils (Urakawa *et al.*, 2016). Since the soil environment had lower nitrate concentrations than arable soils, which were supplemented with higher amounts of nitrogen fertilizers, we considered forest soil to be typically oligotrophic. Surface soil (0–10‍ ‍cm) samples were collected from five randomly selected points at the bottom of a 100-m slope at an altitude of 160 m. After being sieved through a 4-mm mesh sieve to eliminate plant debris and small stones, the soil samples collected were stored at 4°C in the dark until experimentation. Equal weights of soil samples collected at the five sites were evenly mixed, and 1‍ ‍g of the mixed soil sample was used for the most probable number (MPN) enumeration of nitrifiers.

### Medium

Basal medium C described by Suwa *et al.* (1994) was used. Solution A comprising 725‍ ‍mg KH_2_PO_4_ and 1,136‍ ‍mg Na_2_HPO_4_ in 700‍ ‍mL of RO water was amended with either urea or nitrite as the electron donor at various concentrations, the pH of which was adjusted to 7.6 by adding 0.47 M Na_2_CO_3_. Solution B comprised 50‍ ‍mg MgSO_4_·7H_2_O and 20‍ ‍mg CaCl_2_·2H_2_O in 300‍ ‍mL of RO water. Solutions A and B were autoclaved separately at 121°C for 15‍ ‍min and then cooled to room temperature. These two solutions were mixed aseptically, and 0.5‍ ‍mL of sterile 2‍ ‍g‍ ‍L^–1^ Fe-EDDHA and 0.6‍ ‍mL of sterile Trace Element Mixture (ATCC medium #1573) were amended aseptically.

### Measurement of NO_2_^–^ and NO_3_^–^ concentrations

NO_2_^–^ concentrations were assessed by colorimetry with Griess reagent at 538‍ ‍nm absorbance using a multimode plate reader (EnSpire, Perkin Elmer) or a spectrophotometer (Ultrospec 3100 pro; Biochrom). NO_3_^–^ concentrations were measured by the modified Miranda’s method (Miranda *et al.*, 2001), in which NO_3_^–^ was reduced to NO_2_^–^ with vanadium salt. In this procedure, NO_3_^–^ may be overreduced partly to NO, which may result in the underestimation of NO_3_^–^. To prevent the overreduction of NO_3_^–^ to NO, NO_2_^–^ in the sample and that derived from NO_3_^–^ by vanadium reduction were completely and immediately diazotized with Griess reagent. This was achieved by adding Griess reagent to the sample solution before the vanadium solution. To avoid the consumption of the limited volume of the culture, we did not measure urea or ammonium concentrations during the incubation.

### MPN enumeration

A soil sample (1‍ ‍g wet weight) was vigorously suspended in 9‍ ‍mL of basal medium C for 2‍ ‍min to prepare a 10-fold diluted suspension, and 2‍ ‍mL was then transferred to 18‍ ‍mL of basal medium C in a large test tube (φ 22×195‍ ‍mm) and vigorously suspended to prepare a 100-fold diluted soil suspension. This operation was repeated to conduct serial dilutions and prepare 10^1^–10^6^-fold diluted suspensions. In 50 small test tubes containing 4.5‍ ‍mL of medium C containing 0.75‍ ‍mM urea, 0.5‍ ‍mL of each serially diluted suspension (10^2^–10^6^-fold) was inoculated into 10 replicate tubes per dilution. After being cultivated in the dark at 25°C for 9‍ ‍weeks, 100‍ ‍μL of each culture fluid in the tubes was transferred to a well in a 96-well microtiter plate. NO_2_^–^ and NO_3_^–^ concentrations were measured calorimetrically. A culture in which the concentration of NO_3_^–^ was evaluated as >10‍ ‍μM was judged to be positive for NOB. One of the NOB-positive tubes was transferred to 18‍ ‍mL of medium C containing 0.75‍ ‍mM urea and cultivated. Moreover, 10% (v/v) of the culture was transferred to fresh medium every 3‍ ‍weeks to ensure successful subcultures.

### Isolation via the dilution-extinction procedure

Two milliliters of the subculture was diluted 10^6^-fold with medium C containing 1‍ ‍mM nitrite, and 0.5‍ ‍mL of each suspension was dispensed in 192 (first attempt) to 200 (second and third attempts) small test tubes. Aluminum foil was doubly covered on all test tube mouths in a test tube stand, which was placed in a moist chamber and cultivated at 25°C in the dark without shaking. After 6‍ ‍weeks, 100‍ ‍μL of the culture was transferred from each test tube to a 96-well microtiter plate. If at least 1‍ ‍mM NO_2_^–^ was consumed and the same amount of NO_3_^–^ was concomitantly produced, the subculture was positive for NOB. One of the NOB-positive tubes was selected and purified three times via the same dilution-extinction procedure on the same medium.

### Heterotrophic cultivation for a purity check

To check heterotrophic growth, two types of heterotrophic liquid media, diluted tryptic soy broth (TSB/4) (Difco) and fluidized thioglycolate (FTG/2) (Difco) medium, were used (Suwa *et al.*, 1994). Following the addition of 4.5‍ ‍mL each of TSB/4 or FTG/2 into small test tubes, test tubes were capped with aluminum foil and autoclaved at 121°C for 15‍ ‍min. Five hundred microliters of the culture fluid of well-grown “candidate culture CN101” on medium C containing 1‍ ‍mM nitrite was inoculated into each medium and cultivated at 25°C. After a 1-week incubation, we investigated whether heterotrophic growth was visually observed in the culture.

### PCR cloning for a purity check

Bacterial 16S rRNA gene sequences were amplified by PCR with the primer set 27f/1492r (Table S1) and TaKaRa Ex Taq (TaKaRa Bio) with the following thermal protocol: at 94°C for 30‍ ‍s; 35 cycles at 98°C for 10‍ ‍s, 56°C for 30‍ ‍s, and 72°C for 60 s; and at 72°C for 5‍ ‍min. The PCR product was directly ligated into the pCR^TM^4-TOPO^®^ TA vector (Invitrogen) and transformed into One Shot^TM^ TOP10 chemically competent *Escherichia coli*. Clone insert 16S rRNA genes were sequenced by Fasmac.

### Phylogenetic ana­lysis

The bacterial 16S rRNA gene sequence (1,486 bp) of strain CN101 was compared to those of *Nitrobacter* spp. described in a previous study (Vanparys *et al.*, 2007). The phylogenetic tree of the 16S rRNA gene was produced based on Molecular Genetics Analysis version 10.2.1 (MEGAX) (Kumar *et al.*, 2018) and constructed by the maximum likelihood algorithm.

### Fed-batch cultivation

To obtain a sufficient biomass of strain CN101 for physiological and genetic characterization, fed-batch cultivation was performed. As the nitrite concentration reached 20‍ ‍mM, nitrite was repeatedly added to 2.5 L of basal medium C in a 5-L Erlenmeyer flask. The culture was stirred at 100 to 200‍ ‍rpm with a magnet stirrer at 25°C in the dark.

### Optimum temperature

As a preculture, 100‍ ‍mL of strain CN101 containing 1‍ ‍mM nitrite was cultured in a 200-mL flask. In the early stationary phase, 5‍ ‍mL of this preculture was transferred to 45‍ ‍mL of medium C containing 1‍ ‍mM nitrite in a 100-mL flask and incubated at 8, 16, 20, 25, 30, and 35°C in the dark. Each culture was incubated in triplicate. Nitrite and nitrate concentrations were measured over time using the methods described above. Nitrate production rates for 3 days were estimated.

### Protein concentration as the NOB biomass

As an index of the biomass of NOB cells, the protein concentration (μg mL^–1^) of strain CN101 was measured. The cell suspension was separately used to measure kinetics and extract protein. One milliliter of the cell suspension was transferred to a 1.5-mL tube and centrifuged at 4°C for 10‍ ‍min (15,000‍ ‍rpm) to remove the supernatant. The pellet was frozen at –20°C for protein extraction. The protein concentration (μg mL^–1^) was measured using the TaKaRa Bicinchoninic Acid (BCA) Protein Assay Kit (TaKaRa).

### NO_2_^–^ oxidation kinetics

The nitrite oxidation rate was assessed using the cell suspension of strain CN101 amended with 14, 21, 28, or 35‍ ‍μM nitrite (final concentration). Before initiating nitrite oxidation by the addition of nitrite, 12‍ ‍mL of the cell suspension (OD_600_=0.010) in a 50-mL Erlenmeyer flask was incubated in a water bath at 26°C for several min, with stirring at 100‍ ‍rpm with a magnetic stirrer, to equilibrate to the surrounding temperature. After adding a small amount of 1‍ ‍mM NaNO_2_ to initiate nitrite oxidation, 75‍ ‍μL of the cell suspension was sampled, defined as time zero. The cell suspension incubated with nitrite was sampled over time (every 2‍ ‍min four or five more times). Samples were immediately reacted with 75‍ ‍μL of Griess reagent to diazotize all nitrite and stop the nitrite oxidation reaction by NOB, and nitrite concentrations were measured calorimetrically. Nitrite concentrations linearly decreased after a 10-min incubation, and the initial nitrite oxidation rate was assessed as the slope after a linear regression because initial velocity was assumed in this period. *K*_m_ and *V*_max_ values were obtained from the x- and y-intercepts of a Lineweaver-Burk plot of the initial nitrite oxidation rate against the initial nitrite concentration.

### Genome ana­lysis

DNA extraction, DNA sequencing, and assembly and manual curation of the genome of CN101 were conducted at Bioengineering Lab. Briefly, genomic DNA was extracted from strain CN101 using Genomic-tip 100G (QIAGEN) and then sequenced by DNBSEQ-G400 (MGI Tech) for a short read and GridION X5 (Oxford Nanopore Technologies) for a long read. The hybrid assembly of sequences was conducted with the default settings of Unicycler (ver 0.4.7). The completeness and contamination of assembled genome data were confirmed with CheckM ver. 1.1.2. The prediction and annotation of the reconstructed genome were performed using the MicroScope Microbial Genome Annotation & Analysis Platform (MicroScope) and DNA Data Bank of Japan (DDBJ) Fast Annotation and Submission Tool. Orthologous genes were identified using the “Pan/Core-genome” tool in “Comparative Genomics” in MicroScope. Average nucleotide identity (ANI) among *Nitrobacter* genome sequences was calculated with default settings in MicroScope.

### Data availability statement

Data in the present study were deposited in the DDBJ database under the BioProject PRJDB17632.

## Results and Discussion

### MPN enumeration

NO_2_^–^ and NO_3_^–^ in culture tubes for MPN enumeration were exami­ned (Table 1). NO_2_^–^ was not detected, whereas NO_3_^–^ was present in all culture tubes inoculated with 10^2^-fold diluted suspensions. NO_3_^–^ was detected in only 1 of the 10 culture tubes inoculated with 10^3^-fold diluted suspensions, whereas NO_2_^–^ was absent. The MPN of NOB was estimated as 6.58×10^2^ MPN (g wet soil)^–1^.

Aliquots (2‍ ‍mL) from each of the 11 NOB-positive tubes, in which NO_3_^–^ was detected, were transferred to 18‍ ‍mL of freshly prepared medium C containing 0.75‍ ‍mM urea, and cultured at 25°C without shaking. This subculturing procedure was repeated every 3‍ ‍weeks several times. One of the subcultures was selected as the source for isolating NOB.

### Isolation via the dilution-extinction procedure

A 10^6^-fold dilution of the source culture using basal medium C was prepared. A 0.5-mL aliquot of the suspension was transferred to 192 culture tubes containing 4.5‍ ‍mL of medium C with 1.5‍ ‍mM nitrite and was then incubated at 25°C in the dark for 6‍ ‍weeks. Among 192 culture tubes, NO_2_^–^ was completely consumed and NO_3_^–^ accumulated in 6‍ ‍tubes (Table S2). One of the six tubes was selected to further purify the NOB culture, and the same dilution-extinction procedure was repeated using 200 replicate culture tubes. All 14 NOB-positive cultures were subcultured several times every 3‍ ‍weeks to select subcultures capable of stably maintaining nitrite oxidation. One stably maintained culture was selected for purification with the same dilution-extinction procedure, and six NOB-positive cultures were obtained (Table S2).

### Purity check

One of the six NOB-positive cultures was arbitrarily selected to examine whether it was pure with heterotrophic cultivation and PCR cloning. In heterotrophic cultivation, no appreciable growth was observed on four-fold diluted TSB/4 (Difco) or two-fold diluted FTG/2 medium (Difco). In PCR cloning, nucleotide sequences for 109 clones of the amplicon of almost the entire 16S rRNA gene derived from its specific primer were virtually identical: the base sequences of 67 clones were perfectly matched. Of the remaining 42 clones, the base sequences of 31, 9, and 2 clones had 1, 2, and 3 mismatched bases, respectively. It is important to note that all mismatched bases were found in random positions, and no constrained shape was observed in the sequencing chart. Mismatches in the nucleotide sequences of all 109 clones exami­ned may be derived from the inaccuracy of polymerase chain reaction (PCR) amplification and/or cloning. The NOB culture exami­ned for purity, as described herewith, was a pure culture.

### Phylogeny of the 16S rRNA gene

**Fig. 1.**
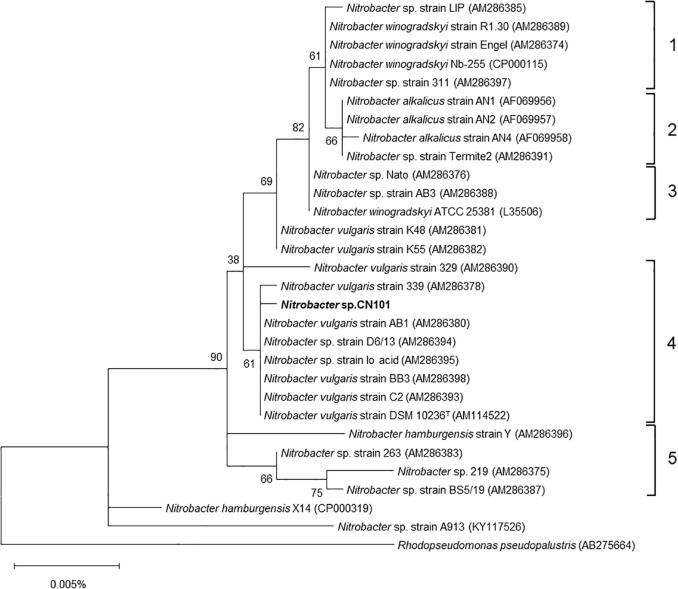
Phylogenetic tree based on 16S rRNA gene sequences of *Nitrobacter* sp. CN101 (1,486 bp) and relative *Nitrobacter* sp. strains. The tree was constructed using the maximum likelihood method (MEGAX version 10.2.1; 1,000 bootstrap iterations). Numbers at the branch nodes are bootstrap values. The scale bar corresponds to 0.005% estimated sequence divergence. Cluster numbers 1 to 5 refer to a previous study (Vanparys *et al.*, 2007). *Nitrobacter* sp. CN101 in the present study is shown in bold and belongs to cluster 4.

The nucleotide sequence (1,486 bp) of the 16S rRNA gene of the isolate was compared to those of nitrifying bacteria, including 28 previously studied strains of the genus *Nitrobacter* (Fig. 1). The isolate was identified as the genus *Nitrobacter* classified as Cluster 4, one of five previously proposed clusters (Vanparys *et al.*, 2007). Based on this result, the isolate was designated as *Nitrobacter* sp. strain CN101. A BLAST search showed that strain CN101 was the most homologous (99.93%) to *Nitrobacter* sp. strain Io acid.

### Obtaining a dense culture of strain CN101 by fed-batch cultivation

**Fig. 2.**
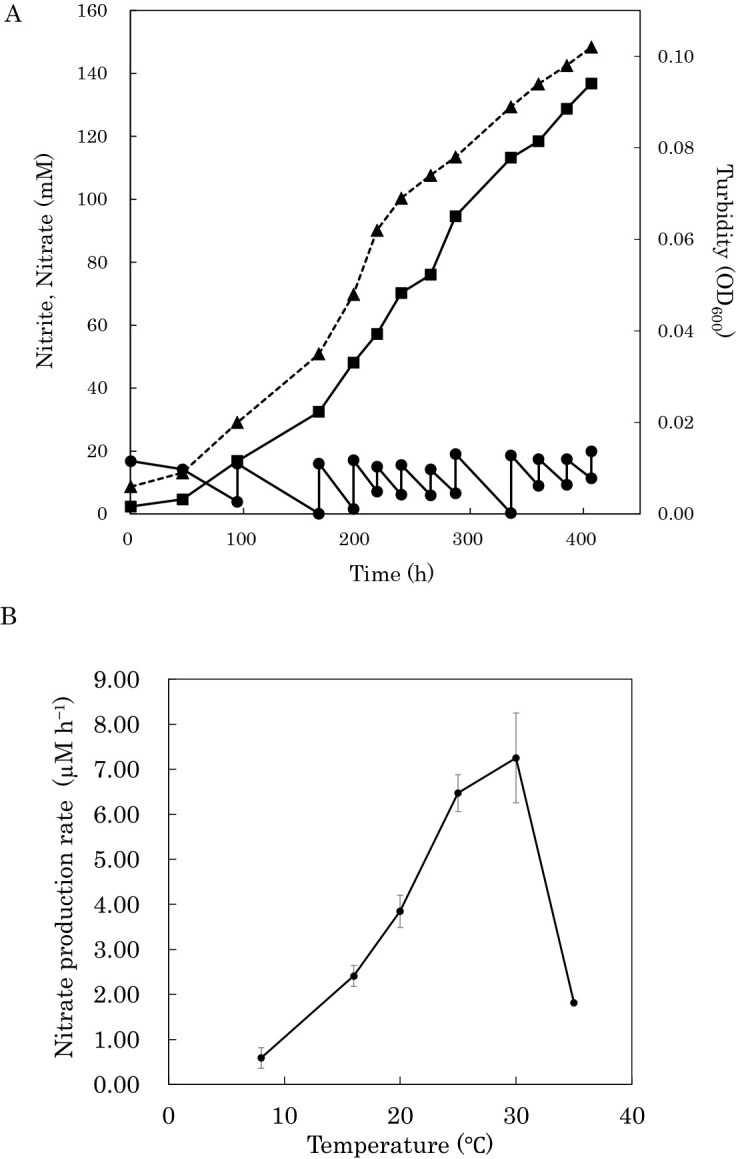
(A) Fed-batch cultivation to obtain the biomass of *Nitrobacter* sp. CN101. Nitrite concentration (circle), nitrate concentration (square), and turbidity (triangle). (B) Optimum temperature of strain CN101. Error bars show biological replicates (*n*=3) and are not shown if smaller than symbols.

Strain CN101 was cultivated on medium C containing 20‍ ‍mM nitrite, and when nitrite was consumed, nitrite was replenished to approximately 20‍ ‍mM by the fed-batch mode of cultivation. Cell density (OD_600_) increased exponentially up to 218‍ ‍h of incubation (Fig. 2A). Nitrite oxidation and OD_600_ then increased linearly as fed-batch cultivation continued. At the end of the experiment after 407‍ ‍h of cultivation, OD_600_ reached 0.102 (Fig. 2A and S2).

### Optimum temperature

Nitrate production rates by strain CN101 were measured under various temperature conditions (Fig. 2B). Strain CN101 showed an optimal temperature of between 25 and 30°C, with almost no nitrate production being observed at 8 or 35°C.

### Kinetics of strain CN101

**Fig. 3.**
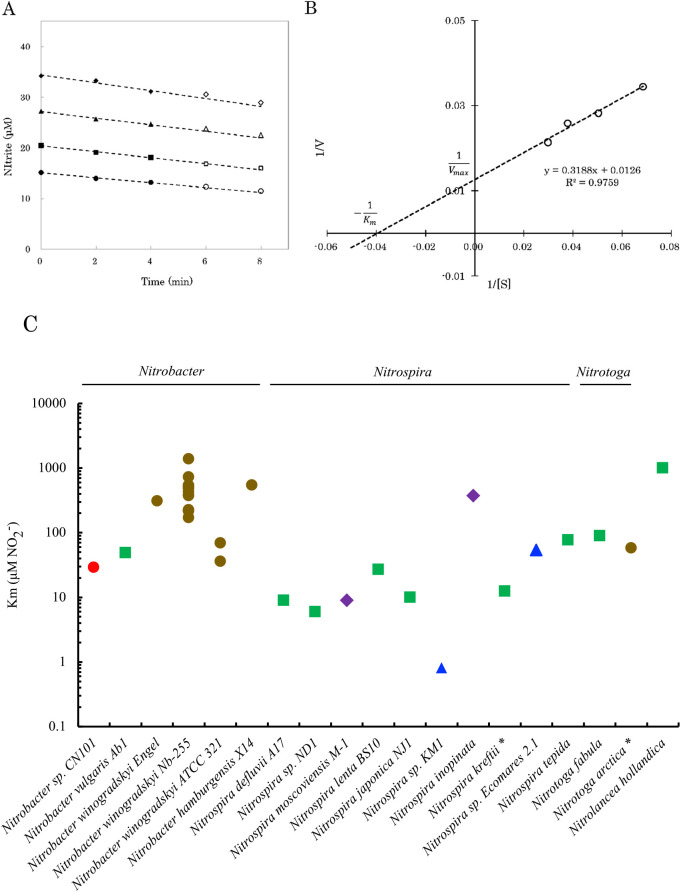
Nitrite oxidation kinetics of *Nitrobacter* sp. CN101. (A) Time course of nitrite consumption. Initial concentrations were adjusted to 14, 21, 28, and 35‍ ‍μM. After suspension in 1‍ ‍mM nitrite and stirring for 5‍ ‍min, cell suspensions were inoculated at 75‍ ‍μL every 2‍ ‍min. Filled symbols were used for a linear regression. (B) Double reciprocal plot of the rate constant and nitrite concentration. (C) Comparison of affinities for NO_2_^–^ between *Nitrobacter* sp. CN101 and previously reported pure cultures of NOB (*Nitrobacter*, *Nitrospira*, *Nitrotoga*, and *Nitrolancea*), except for two enrichment cultures marked with asterisks. Cultures were derived from soil (circles), activated sludge (squares), artificial freshwater (triangles), and a biofilm on a metal pipe (diamonds).

The nitrite oxidation rate of strain CN101 was measured using 14, 21, 28, and 35‍ ‍μM nitrite (Fig. 3A). Nitrite decreased linearly over time. The slope of the line was selected as the zero-order rate constant using the first three points of the line because no greater than 15% of the initial nitrite concentration was oxidized in the first three points, which may be regarded as the initial rate of nitrite oxidation. *V*_max_, the maximum nitrite oxidation rate, and *K*_m_, the half-saturation constant, were measured from the double reciprocal plot of the concentration of nitrite and initial nitrite oxidation rate (Fig. 3B). Consequently, *V*_max_ was 79.4‍ ‍μmol NO_2_^–^ mg^–1^ protein h^–1^ and *K*_m_ was 25.3‍ ‍μM NO_2_^–^. To confirm the reproducibility of the kinetics experiment, it was reimplemented using the same culture. The data obtained showed that *V*_max_ was 78.1‍ ‍μmol NO_2_^–^ mg^–1^ protein h^–1^ and *K*_m_ was 32.6‍ ‍μM NO_2_^–^ (Fig. S3). The *K*_m_ value for *Nitrobacter* sp. CN101 was compared to those previously reported for strains of the genera *Nitrobacter* (Both *et al.*, 1992; Hunik *et al.*, 1993; Nowka *et al.*, 2015a), *Nitrospira* (Nowka *et al.*, 2015a; Kits *et al.*, 2017; Ushiki *et al.*, 2017; Jacob *et al.*, 2017; Fujitani *et al.*, 2020; Sakoula *et al.*, 2021), *Nitrotoga* (Nowka *et al.*, 2015a; Kitzinger *et al.*, 2018; Keuter *et al.*, 2023), and *Nitrolancea* (Sorokin *et al.*, 2012) (Fig. 3C).

The *K*_m_ value of strain CN101 was the lowest among those obtained for previous *Nitrobacter* strains, which was close to those for *Nitrospira* strains, suggesting that *Nitrobacter* strains do not always exhibit lower affinity for nitrite than other NOB. *Nitrobacter* strains may sometimes be competitive for nitrite with other NOB in natural environments. Conventional mole­cular approaches and potential activity tests revealed that major NOB in soils were the genera *Nitrobacter* and *Nitrospira* (Attard *et al.*, 2010). In the soil NOB community, the abundance of *Nitrobacter* and *Nitrospira* may be affected by nitrogen levels and its availability. In forest and agricultural soils with high nitrogen availability or long-term fertilization, *Nitrobacter*-like NOB abundance was shown to exceed that of *Nitrospira*-like NOB (Wertz *et al.*, 2012; Wang *et al.*, 2014). According to previous studies on aquatic environments and wastewater treatment plants (Schramm *et al.*, 1999; Wagner *et al.*, 2002; Blackburne *et al.*, 2007), *Nitrobacter* had a higher growth rate and lower affinity for nitrite, whereas *Nitrospira* had a lower growth rate and higher affinity for nitrite. In the present study, *Nitrobacter*-like bacterial enrichment was achieved using media containing urea as an energy source. Consequently, an oligotrophic *Nitrobacter* strain with a higher affinity for nitrite was isolated. This result suggests that different *Nitrobacter* species adapt to different nitrite concentrations in various soil environments.

### Genome properties of strain CN101 and insights from comparative genomics

The genomic information of *Nitrobacter* sp. strain CN101 was summarized and compared to three representative strains of the genus *Nitrobacter*: *Nitrobacter winogradskyi* Nb-255 (Starkenburg *et al.*, 2006), *Nitrobacter hamburgensis* X14 (Starkenburg *et al.*, 2008), and *Nitrobacter vulgaris* AB1 (Mellbye *et al.*, 2017). The reconstructed complete genome sequence of strain CN101 was 4,315,382 bp long with a 59.56% GC content, 5041 predicted CDS, 57 tRNA, and 3 rRNA. Additionally, strain CN101 had no plasmids. We confirmed the purity of strain CN101 from whole genome sequencing data and no microbial contamination was detected. Core and pan genome ana­lyses of the four *Nitrobacter* strains revealed high genomic similarities (Fig. S4). Moreover, an ANI ana­lysis of *Nitrobacter* genome sequences revealed that strain CN101 showed close genetic relatedness to *N. vulgaris* AB1 (96.1%). The two strains also showed high similarity (99.93%) at the 16S rRNA sequence level. Collectively, these results support the identification of our isolate as *N. vulgaris*.

**Fig. 4.**
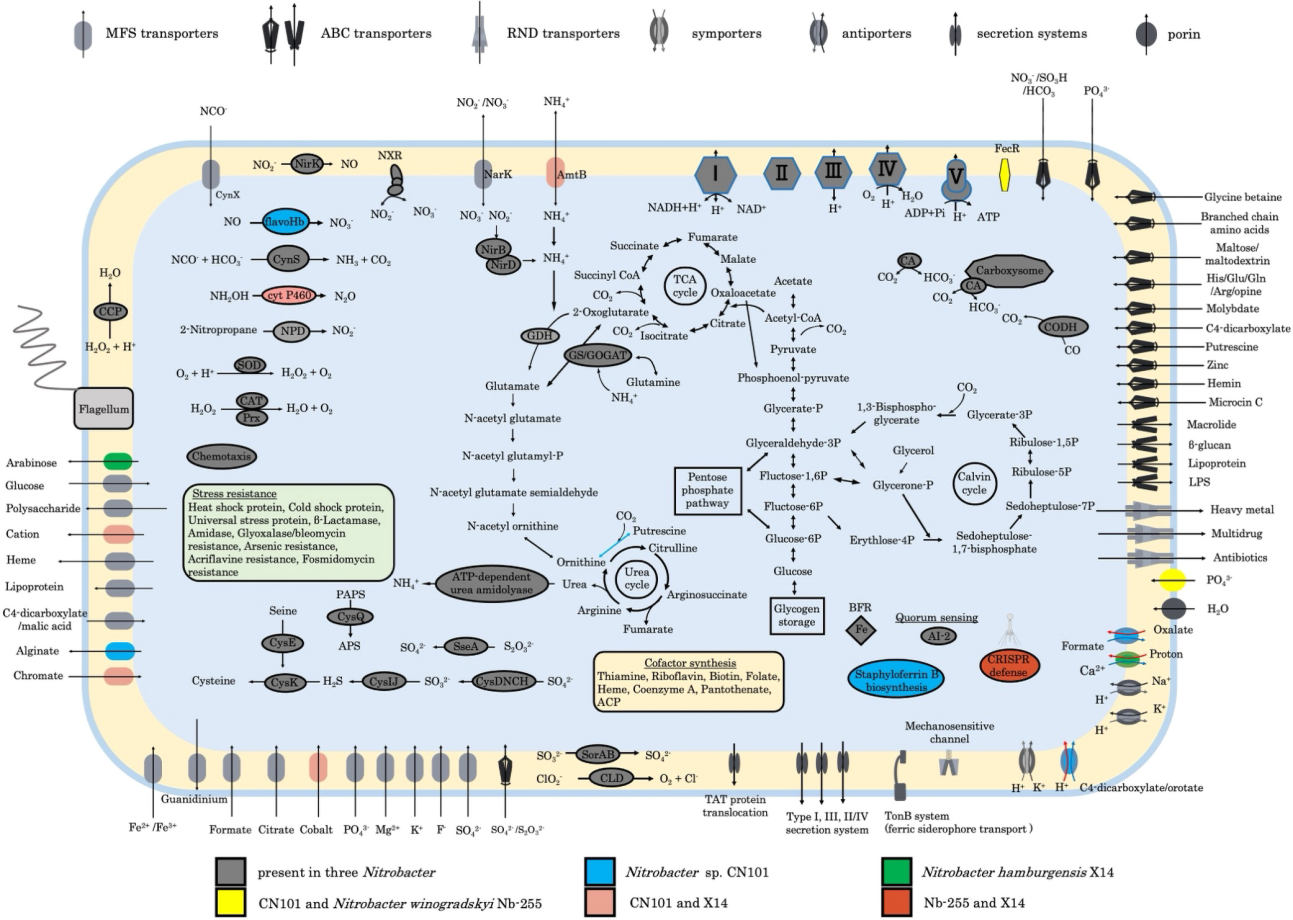
Schematic representation of a cell showing key and unique metabolic features encoded by genomes of *Nitrobacter* sp. CN101, *N. winogradskyi *Nb-255, and *N. hamburgensis* X14. Core functions shared in three *Nitrobacter* species are shown in gray, specific features of *Nitrobacter* sp. CN101 are shown in blue, and those of *N. hamburgensis* X14 are shown in green. Specific features shared in two *Nitrobacter* are shown in yellow (*Nitrobacter* sp. CN101 and *N. winogradskyi* Nb-255), pink (*Nitrobacter* sp. CN101 and *N. hamburgensis* X14), and red (*N. winogradskyi* Nb-255 and *N. hamburgensis* X14). There were no specific features of only *N. winogradskyi *Nb-255. Enzyme complexes of the electron transport chain are labeled by Roman numerals. AI-2, autoinducer; AmtB, ammonium transporter; BFR, bacterioferritin; CA, carbonic anhydrase; CAT, catalase; CCP, cytochrome *c* peroxidase; CLD, chlorite dismutase; CODH, carbon monoxide dehydrogenase; CRISPR, clustered regularly interspaced short palindromic repeats; flavoHb, flavohemoglobin; GDH, glutamate dehydrogenase; GS/GOGAT, glutamine synthetase/glutamate synthase; LPS, lipopolysaccharide; NarK, nitrite/nitrate transporter; NirBD, nitrite reductase; NirK, copper-containing nitrite reductase; NPD, nitropropane dioxygenase; Prx, peroxidase; SorAB, cytochrome *c* sulfite oxidoreductase; SOD, superoxide dismutase; SseA, thiosulfate sulfurtransferase.

We subsequently selected the two complete genomes of *N. winogradskyi* Nb-255 and *N. hamburgensis* X14, both isolated from soil, for comparisons of metabolic features with strain CN101. *N. vulgaris* AB1 was not selected because its genome was not complete and its isolation source was sewage. The predicted metabolic features encoded by the genome of strain CN101 were schematically represented and compared to *N. winogradskyi* Nb-255 and *N. hamburgensis* X14 isolated from soil (Fig. 4). Nitrite oxidoreductase (NXR) of *Nitrobacter* comprises subunit α (NxrA), subunit β (NxrB), and subunit γ (NxrG), and is phylogenetically close to Nar-type nitrate reductase. Strain CN101 possessed more *narK* genes related to nitrite/nitrate transport than the other *Nitrobacter* strains. A previous study showed that *narK1* exhibited higher affinity for nitrate than *narK2* in *Pseudomonas aeruginosa* strain PaO1 (Sharma *et al.*, 2006). Since multiple *narK* genes are related to different affinities for nitrite/nitrate, strain CN101 may be more competitive for nitrite/nitrate availability than other NOB. Regarding nitrite reductase, strain CN101 was found to only have one copy of the *nirK* gene cluster (NvuCN101_1165–NvuCN101_1170), a copper-containing nitrite reductase reducing nitrite to nitric oxide under anaerobic conditions (Table S3). NirK was previously shown to play a role in the removal of nitrite in the periplasm under anaerobic conditions (Starkenburg *et al.*, 2008). Moreover, strain CN101 possessed one copy of the *nirBD* genes that reduce nitrite to NH_4_^+^ (NvuCN101_3722–NvuCN101_3723), cyanate transporters (NvuCN101_2749 and NvuCN101_3767), and cyanate hydratase that turns cyanate into ammonia (NvuCN101_3232) (Table S3), which may be used for nitrogen assimilatory metabolism by strain CN101.

Among other nitrogen metabolism genes, strain CN101 possessed nitric oxide dioxygenase (flavohemoglobin; Ncn_0698), which converts NO into NO_3_^–^ under aerobic conditions (Gardner *et al.*, 1998; Frey and Kallio, 2003), whereas the other two *Nitrobacter* strains did not have genes related to flavohemoglobin. All three *Nitrobacter* strains possessed ATP-dependent urea amidolyase (NvuCN101_2800–NvuCN101_2801), which converts urea into NH_4_^+^ (Table S3). However, this enzyme is dependent on ATP and is inefficient for energy metabolism. Additionally, ATP-independent urea amidolyase (urease) and urea transporters were not detected among the three *Nitrobacter* strains. Therefore, urea outside of the cell may not be utilized. Strain CN101 and *N. hamburgensis* X14 possessed cytochrome *P460* genes (NvuCN101_0829 and NvuCN101_3969 in Table S3), similar to the genes of cytochrome *P460* in the genus *Bradyrhizobium*. A previous study demonstrated that cytochrome *P460* in AOB combined with NO and changed NH_2_OH into N_2_O (Caranto *et al.*, 2016). Although the NH_3_/NH_4_^+^ transporter described above may be functioning as a transporter of NH_2_OH into cells (Hanson *et al.*, 2013), the metabolism of N_2_O generated by cytochrome *P460* remains unclear.

### Ecological aspect of *N. vulgaris*

In Latin ‘vulgaris’ means ‘common’. Strains identified as *N. vulgaris* have been isolated from various environments, including soils and freshwater (Bock *et al.*, 1990). Therefore, a member of *N. vulgaris* may be dominant over other *Nitrobacter* species in environments. The generation time of strain CN101 calculated based on nitrate production was 34.5‍ ‍h (data not shown). A previous kinetics experiment revealed that *N. vulgaris* AB1 exhibited the highest nitrite affinity and maximum cell activity among the *Nitrobacter* species investigated (Nowka *et al.*, 2015a). Based on this finding, a member of *N. vulgaris* including strains CN101 and AB1 may adapt to lower nitrite concentrations in oligotrophic environments than other *Nitrobacter* species. Furthermore, we exami­ned common genomic properties among *N. vulgaris* and found that *N. vulgaris* AB1 and *N. vulgaris* DSM10236 (Soghomonian *et al.*, 2024) possessed multiple copies of the *narK* gene as a nitrite/nitrate transporter, similar to strain CN101. This result suggests that *N. vulgaris* increases nitrite availability and is widespread in environments. However, the kinetics of the *narK* gene among *Nitrobacter* species remains unknown and warrant further investigation. Additionally, the use of metagenomic data from DNA databases will be beneficial for understanding the environmental distribution and abundance of *Nitrobacter* species, including *N. vulgaris*.

## Conclusion

In the present study, oligotrophic nitrite-oxidizing *Nitrobacter* were initially enriched using a medium containing urea and then isolated by subculturing in a medium with nitrite and performing the extinction-dilution procedure. Strain CN101 exhibited higher affinity for nitrite than other previously reported *Nitrobacter* strains. Furthermore, the genome of strain CN101 possessed more nitrite/nitrate transporters, which may flexibly adapt to varying nitrite concentrations in soils. Previously isolated *Nitrobacter* strains also showed physiological and genomic diversity. Due to the importance of these results, the potential contribution of *Nitrobacter* species may still be underestimated. Therefore, obtaining and characterizing further *Nitrobacter* isolates will improve our ecological understanding of the genus *Nitrobacter* as a major NOB.

## Citation

Kobayashi, Y., Ninomiya, T., Shiraishi, Y., Kaneko, A., Kuroiwa, M., Suwa, Y., and Fujitani, H. (2025) Physiological and Genomic Characterization of Oligotrophic *Nitrobacter* Isolated from a Forest Soil in Japan. *Microbes Environ ***40**: ME24114.

https://doi.org/10.1264/jsme2.ME24114

## Supplementary Material

Supplementary Material

## Figures and Tables

**Table 1.** MPN score of nitrate production after 9‍ ‍weeks of cultivation at 25°C of a forest soil sample on a medium containing 0.75‍ ‍mM urea. One of the tubes marked in plus was selected as the isolation source of NOB.

Dilution	MPN score*^a^*									
10^–2^	+	+	+	+	+	+	+	+	+	+
10^–3^	–	–	–	–	+	–	–	–	–	–
10^–4^	–	–	–	–	–	–	–	–	–	–
MPN ([g wet soil]^–1^) of NOB*^b^*	6.58×10^2^									

^a^ +: nitrate was produced. No nitrite accumulated. ^b^ MPN estimates for NOB were based on the number of tubes in which nitrate production was detected.
