# Supplementary material for: Physiological and Genomic Characterization of Oligotrophic Nitrobacter Isolated from a Forest Soil in Japan
Source: Microbes Environ. 2025 Jun 17;40(2):ME24114. doi: 10.1264/jsme2.ME24114 (PMC12213059; doi:10.1264/jsme2.ME24114)
Supplement: Supplementary file 1 — Supplementary Material [file 40_24114_s1.pdf]

Supplemental Material

Fig. S1

(A) Soil samples were collected from 0-10 cm soil layer at the bottom (0-30 m) of a mountain slope on a straight line (average slope; 29°, a distance; 100 m, an altitude; 160-230 m) at Chiba Forestry Experimental Station managed by the University of Tokyo on July 29, 2016. (B) Phot image of sampling site (soil; cambisols, vegetation; cedar and cypress).

Fig. S2

Biomass of *Nitrobacter* sp. CN101 with high cell density. Cultivation start (left), 407 hours later (right). Optical density was 0.102 at 407 hours of cultivation.

Fig. S3

Nitrite oxidation kinetics of *Nitrobacter* sp. strain CN101. The kinetics experiment was reimplemented using the same culture shown in Fig. 3 (A) Time course of nitrite consumption. Initial concentrations were adjusted 14, 21, 28, 35 μM. After suspended 1 mM nitrite and stirred 5 minutes, cell suspensions were inoculated 75 μL every 2 minutes. Filled symbols were used for linear regression. (B) double reciprocal plot of rate constant and nitrite concentration.

Fig. S4

Comparative genomics of four *Nitrobacter* strains. Orthologous genes were clustered by "Pan/Core-genome" tool in MicroScope. The numbers indicated the shared orthologous clusters among four strains.

Table S1

Primers for amplified 16S rRNA of strain CN101 used in this study.

Table S2

Dilution-extinction was conducted 3 times using inorganic medium containing 1 mM NO_2^-.

Table S3

Comparison of genes responsible for nitrogen metabolism and transport in *Nitrobacter*.

References

Lane DJ. 16S/23S rRNA sequencing. In: Stackebrandt E, Goodfellow M (eds). *Nucleic Acid Techniques in Bacterial Systematics*. Chichester, United Kingdom: Wiley, 1991;115–75.

Fig. S1

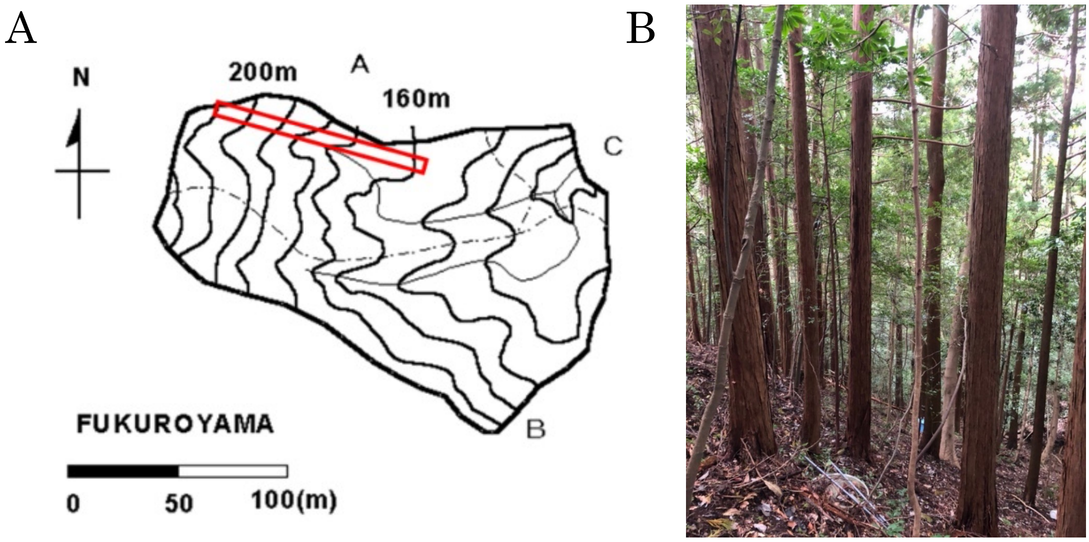

Fig. S2

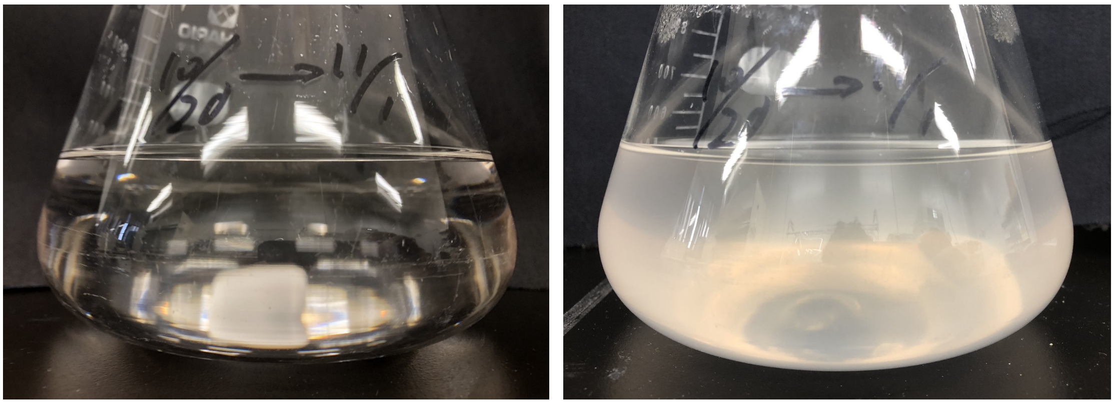

Fig. S3

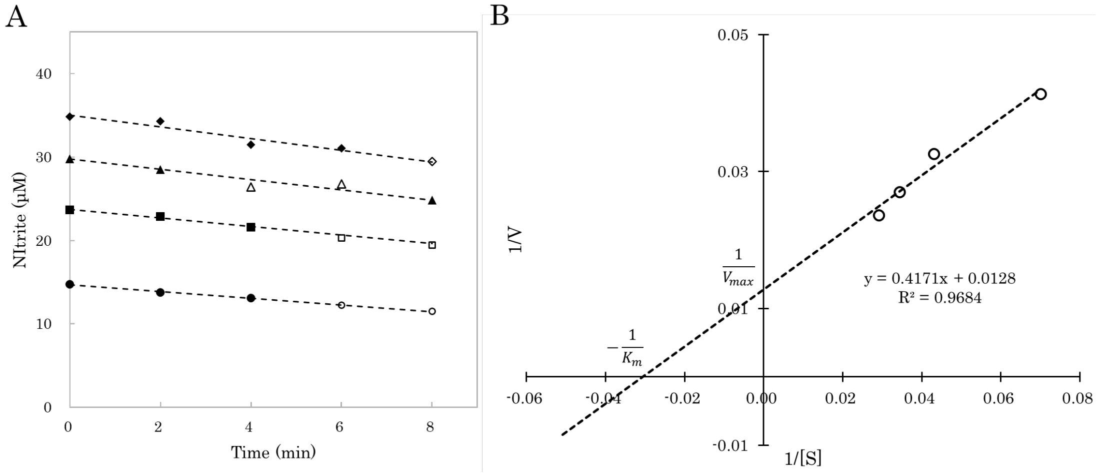

A
40
30
20
10
0
NItrite (μM)
0
2
4
6
8
Time (min)
B
0.05
0.03
0.01
-0.01
1/V
$\frac{1}{V_{max}}$
$-\frac{1}{K_m}$
y = 0.4171x + 0.0128
R² = 0.9684
-0.06
-0.04
-0.02
0.00
0.02
0.04
0.06
0.08
1/[S]

Fig. S4

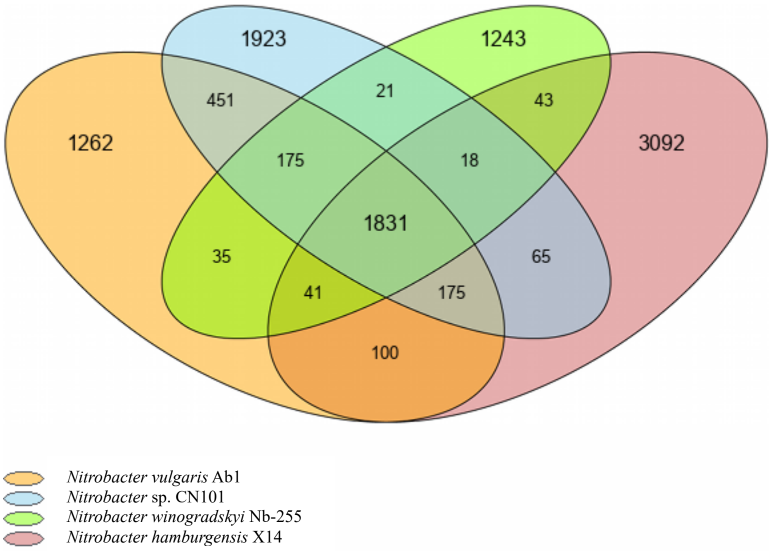
1923
1243
1262
451
21
43
3092
175
18
1831
35
65
41
175
100
Nitrobacter vulgaris Ab1
Nitrobacter sp. CN101
Nitrobacter winogradskyi Nb-255
Nitrobacter hamburgensis X14

Table S1 Primers of 16S rRNA gene

Primer name	Sequence (5’-3’)	Reference
27f	AGA GTT TGA TCM TGG CTC AG	Lane 1991
1492r	TAC GGY TAC CTT GTT ACG ACT T	

Table S2 Dilution-extinction conducted using inorganic medium containing 1 mM NO_2^-

exp	dil	# of aliquots	# of NO_3^- produced
1	10^{-6}	192	6
2	10^{-6}	200	14
3	10^{-6}	200	6

Table S3 Comparison of genes responsible for nitrogen metabolism and transport in *Nitrobacter* .

Category and putative function	Gene(s) in;		
	Nitrobacter sp. CN101	*N. winogradskyi* Nb-255	*N. hamburgensis* X14
NXR ($NO_2^- \leftrightarrow NO_3^-$)			
Hypothetical protein	NvuCN101_3659	Nwi_0772	Nham_3451
Cytochrome *c* , class I	NvuCN101_3658	Nwi_0773	Nham_3450
nxrA ; nitrite oxidoreductase, alpha subunit	NvuCN101_3657	Nwi_0774	Nham_3449
nxrX ; nitrite oxidoreductase, subunit X	NvuCN101_3656	Nwi_0775	Nham_3448
nxrB ; nitrite oxidoreductase, beta subunit	NvuCN101_3655	Nwi_0776	Nham_3447
nxrD ; nitrite oxidoreductase, delta subunit	NvuCN101_3654	Nwi_0777	Nham_3446
nxrG ; nitrite oxidoreductase, gamma subunit	NvuCN101_3653	Nwi_0778	Nham_3445
narKX ; nitrite/nitrate major facilitator superfamily transporter	NvuCN101_3652	Nwi_0779	Nham_3444
TDT family transport protein	NvuCN101_3651	Nwi_0780	Nham_3443
nxrA homolog(s)	NP	Nwi_2068	Nham_0951, Nham_2961
nxrB homolog	NvuCN101_3584	Nwi_0965	Nham_3289
narKX homolog(s)	NvuCN101_1149, NvuCN101_1500, NvuCN101_1646	Nwi_6627	Nham_1444
narK1	NvuCN101_2730	Nwi_1419	Nham_2459
NirK ($NO_2^- \rightarrow NO$)			
nirK ; copper-containing nitrite reductase	NvuCN101_1170	Nwi_2648	Nham_3282
Cytochrome *c* , class IC	NvuCN101_1169	Nwi_2649	Nham_3283
Cytochrome *c* , class I	NvuCN101_1168	Nwi_2650	Nham_3284
Copper oxidase	NvuCN101_1167	Nwi_2651	Nham_3285
Cytochrome *c* biogenesis factor	NvuCN101_1166	Nwi_2652	Nham_3286
nsrR ; transcriptional regulator, BadM/Rrf2 family	NvuCN101_1165	Nwi_2653	Nham_3287
NirBD ($NO_2^- \rightarrow NH_4^+$)			
nirB ; nitrite reductase [NAD(P)H] large subunit	NvuCN101_3723	Nwi_0719	Nham_2965
nirD ; nitrite reductase [NAD(P)H] small subunit	NvuCN101_3722	Nwi_0720	Nham_2964
Nitric oxide denitrification ($NO \rightarrow NO_3^-$)			
Nitric oxide dioxygenase (Flavohemoglobin)	NvuCN101_0630	NP	NP
Nitropropane dioxydenase (nitropropane→NO_2^-)			
NPD ; 2-nitropropane dioxygenase	NvuCN101_4213	Nwi_3001	Nham_1075
Formate and nitrite transporter	NvuCN101_4218	Nwi_3006	Nham_1071
NPD ; 2-nitropropane dioxygenase	NP	NP	Nham_2278
Ammonium transport (NH_4^+(out)→NH_4^+(in))			
amtB ; ammonium transporter	NvuCN101_0229	NP	Nham_0084
glnK ; nitrogen regulatory protein P-II 1	NvuCN101_0230	Nwi_0076	Nham_0085
amtB ; ammonium transporter homolog	NvuCN101_0231	NP	NP
glnK ; nitrogen regulatory protein P-II 1 homolog	NvuCN101_0232	NP	Nham_0086
ATP-dependent urea amidolyase (urea → NH_4^+)			
Allophanate hydrolase subunit 1	NvuCN101_2800	Nwi_1501	Nham_2040
Allophanate hydrolase subunit 2	NvuCN101_2801	Nwi_1502	Nham_2041
5-oxoprolinase component A	NvuCN101_2802	Nwi_1503	Nham_2042
Nitrate/sulfonate/bicarbonate ABC transporter (NO_3^-(out)↔NO_3^-(in))			
Nitrate/sulfonate/bicarbonate ABC transporter periplasmic binding protein	NvuCN101_0437	Nwi_0331	Nham_0425
Nitrate/sulfonate/bicarbonate ABC transporter ATP-binding protein	NvuCN101_0438	Nwi_0332	Nham_0426
Nitrate/sulfonate/bicarbonate ABC transporter ATP-binding protein	NvuCN101_0439	Nwi_0333	Nham_0427
Nitrate/sulfonate/bicarbonate ABC transporter ATP-binding protein	NvuCN101_4357	Nwi_0458	NP
Nitrate/sulfonate/bicarbonate ABC transporter periplasmic binding protein	NvuCN101_4358	Nwi_0457	NP
Nitrate/sulfonate/bicarbonate ABC transporter membrane protein	NvuCN101_4359	Nwi_0456	NP
Nitrate/sulfonate/bicarbonate ABC transporter ATP-binding protein	NP	Nwi_0671	NP
Nitrate/sulfonate/bicarbonate ABC transporter periplasmic binding protein	NP	Nwi_0674	NP
Nitrate/sulfonate/bicarbonate ABC transporter periplasmic binding protein	NP	Nwi_0680	NP
Nitrate/sulfonate/bicarbonate ABC transporter periplasmic binding protein	NP	Nwi_0681	NP
Cyanate hydratase (cyanate→NH_4^+)			
cynS ; cyanate hydratase	NvuCN101_3232	Nwi_1302	Nham_1631
cynX ; cyanate transporter	NvuCN101_2749	Nwi_1437	Nham_1829
cynX homolog	NvuCN101_3767	NP	NP
Cytochrome *P460* ($NH_2OH \rightarrow N_2O$)			
Putative cytochrome *P460*	NvuCN101_3969	NP	NP
Putative cytochrome *P460*	NvuCN101_0829	NP	Nham_2497
Nitroreductase			
Nitroreductase	NvuCN101_2184	Nwi_2036	Nham_1668
Nitroreductase	NP	NP	Nham_1903
Glutamine synthetase (NH_4^+→L-glutamine)			
glnA ; glutamine synthetase	NvuCN101_2287	Nwi_1904	Nham_2234
glnB ; nitrogen regulatory protein PII-1	NvuCN101_2288	Nwi_1903	Nham_2233
GMP synthase (glutamine-hydrolyzing)	NvuCN101_3909	NP	NP
Alcohol dehydrogenase	NvuCN101_3910	NP	NP
Glutamine synthetase	NvuCN101_3911	NP	NP
Glutamate synthase (L-glutamine →L-glutamate)			
gltB ; glutamate synthase subunit	NvuCN101_4160	Nwi_2953	Nham_1138
gltD ; glutamate synthase subunit small chain	NvuCN101_4162	Nwi_2954	Nham_1137
Glutamate dehydrogenase (NH_4^+↔L-glutamate)			
gdhA ; glutamate dehydrogenase	NvuCN101_1951	Nwi_2286	Nham_2702
Glutamate dehydrogenase	NP	NP	Nham_0996
Carbamoyl-phosphate synthase (L-glutamine →L-glutamate)			
carA ; carbamoyl-phosphate synthase (glutamine-hydrolysing)	NvuCN101_1377	Nwi_2446	Nham_2871
Ferritin and Dps	NvuCN101_1376	Nwi_2447	Nham_2872
car B ; carbamoyl-phosphate synthase (glutamine-hydrolysing)	NvuCN101_1374	Nwi_2449	Nham_2875
Nitrogen regulatory protein (histidine kinase)			
ntrB ; sensory histidine kinase/phosphatase	NvuCN101_2758	Nwi_1444	Nham_1836
ntrC ; DNA-binding transcriptional dual regulator	NvuCN101_2759	Nwi_1445	Nham_1837
ntrY ; nitrogen regulatory protein	NvuCN101_2762	Nwi_1446	Nham_1838
nirX ; nitrogen assimilation regulatory protein	NvuCN101_2763	Nwi_1447	Nham_1839

PTSIIA-like nitrogen regulatory protein			
ptsN ; PTSIIA-like nitrogen regulatory protein	NvuCN101_0300	Nwi_0179	Nham_0171
ptsN ; PTSIIA-like nitrogen regulatory protein homolog	NvuCN101_3869	NP	NP

NP, not present.
